# Transvesical Clipless Robot-Assisted Radical Prostatectomy Using a Multiport Platform: Technical Feasibility and Early Outcomes

**DOI:** 10.1590/S1677-5538.IBJU.2025.0762

**Published:** 2026-02-20

**Authors:** Thiago Camelo Mourão, Plínio Ramos Pinto, Jayme Quirino Caon Nobre, Ivan Augusto Agudo Miranda, Eduardo Zanotta Rodrigues, Gustavo de Pádua Brazão, Stênio de Cássio Zequi

**Affiliations:** 1 AC Camargo Cancer Center Departamento de Urologia São Paulo SP Brasil Departamento de Urologia, AC Camargo Cancer Center, São Paulo, SP, Brasil

## Abstract

**Introduction:**

Robot-assisted radical prostatectomy (RARP) continues to evolve with surgical approaches aimed at preserving continence-related anatomy and optimizing postoperative recovery (1). Transvesical RARP has gained increasing interest, particularly with the advent of single-port platforms (2, 3). Building on our prior experience with clipless techniques and functional outcome optimization (4), we evaluated a multiport transvesical approach.

**Objective:**

To describe, step-by-step, a clipless transvesical RARP technique and to report its feasibility and early functional and oncologic outcomes.

**Materials and Methods:**

An intravesical, line-of-sight technique was employed, with abdominal trocars positioned similarly to conventional transperitoneal RARP. The surgical sequence included: posterior release; longitudinal cystotomy with suspension sutures; semicircumferential bladder neck incision; bilateral clipless lateral dissection (blunt and sharp); anterior and apical release with dorsal venous complex control; urethral transection; urethrovesical anastomosis using two 3-0 barbed sutures; and single-layer cystotomy closure. Three consecutive patients with localized prostate cancer and moderate prostate volumes were included.

**Results:**

All procedures were completed as planned. Perioperative morbidity was low; one Clavien–Dindo grade II complication was managed conservatively. Early continence was observed in all patients, including at the first postoperative evaluation (45 days). One patient (Case 3) presented with a focal apical positive surgical margin on final pathology; prostate-specific antigen levels were undetectable in all cases during early follow-up. The approach was technically feasible and familiar to surgeons experienced in anterior RARP. Larger prostate volume may limit intravesical working space and apical visualization.

**Conclusions:**

We describe a reproducible clipless transvesical RARP performed using a multiport platform, addressing a body of literature predominantly focused on single-port systems. The technique demonstrated encouraging early functional outcomes in carefully selected patients (5); however, larger prostate volumes and longer follow-up are required to further define its oncologic and functional durability. This video complements our previous publication on alternative RARP approaches supporting anterior-preserving strategies (6).

**Video 1:** Available at: VIDEO

## Data Availability

Data Not Provided
